# Making the Invisible Visible: Narrative Repair in Representing Experiences of Black Women in Dementia Research

**DOI:** 10.1093/geroni/igaf122.972

**Published:** 2025-12-31

**Authors:** H Shellae Versey

**Affiliations:** Fordham University, New York, New York, United States

## Abstract

This paper draws on research insights and analysis of personal narratives to consider cultural narratives about Black women’s experiences of aging, dementia, and caregiving. Black women face elevated risks for dementia, associated health conditions, and financial vulnerability in later life. Attention to cultural narratives about Black women and aging, as these narratives circulate within health care and within communities, may reveal positive yet unrealistic assumptions about what Black women should be able to endure or manage, for themselves or for others. This paper presents an argument for narrative repair, relevant to dementia research, that recognizes culturally meaningful narrative features concerning strength, while providing better recognition of and support to Black women navigating significant challenges in later life.

